# A complete response to mFOLFOX6 and panitumumab chemotherapy in advanced stage rectal adenocarcinoma: a case report

**DOI:** 10.1186/1477-7819-12-63

**Published:** 2014-03-26

**Authors:** Hiromichi Sonoda, Tomoharu Shimizu, Eiji Mekata, Yoshihiro Endo, Mitsuaki Ishida, Tohru Tani

**Affiliations:** 1 Department of Surgery, Shiga University of Medical Science, Seta Tsukinowa-cho, Otsu, Shiga 520-2192, Japan; 2 Department of Clinical Laboratory Medicine, Shiga University of Medical Science, Seta Tsukinowa-cho, Otsu, Shiga 520-2192, Japan

**Keywords:** Neoadjuvant chemotherapy, Rectal cancer, Panitumumab

## Abstract

**Background:**

Pathological complete remission of advanced stage rectal adenocarcinoma by chemotherapy alone is rare. A case of advanced stage, low-lying rectal adenocarcinoma in which a complete response to treatment was obtained with mFOLFOX6 and panitumumab (Pmab) is reported.

**Case presentation:**

A 53-year-old man was referred to Shiga University of Medical Science hospital Shiga, Japan, complaining of bloody stool. Gastrointestinal endoscopy was performed, and advanced stage rectal adenocarcinoma was diagnosed. Computed tomography (CT) revealed regional lymph node metastases in the mesorectum. Neoadjuvant chemotherapy (NAC) with mFOLFOX6 and Pmab was planned.

Endoscopy following four courses of chemotherapy revealed that the rectal cancer had been markedly reduced, and the results of biopsies of the rectal tumor were negative for cancer. On CT, the mesorectal lymph node metastases had disappeared. Total intersphincteric resection (ISR) with a handsewn coloanal anastomosis was performed. Histological examination showed a complete response to mFOLFOX6 and Pmab in advanced stage rectal cancer.

**Conclusion:**

The result seen in this case suggests that short-term NAC with mFOLFOX6 and Pmab was effective for low-lying rectal adenocarcinoma.

## Background

In Western countries, the standard treatment for advanced stage rectal cancer is neoadjuvant chemoradiotherapy (CRT) followed by surgery [1]. On the other hand, curative surgery including lateral lymph node dissection followed by adjuvant chemotherapy is the standard treatment for advanced rectal cancer in Japan [2]. Recently, neoadjuvant CRT followed by anus-preserving surgery has also been reported in Japan [3]. However, it was reported that postoperative anal function was decreased when the effect of preoperative CRT was strong in patients treated with intersphincteric resection (ISR) [3]. Use of panitumumab (Pmab) and cetuximab (Cmab), anti-endothelial growth factor receptor (EGFR) antibodies, has resulted in earlier tumor shrinkage for *K-ras* wild type metastatic colorectal cancer [4]. The case of a 53-year-old man with stage III low rectal cancer who had a complete response to neoadjuvant oxaliplatin, 5-fluorouracil (5-FU) and l-folinic acid (mFOLFOX6) and Pmab chemotherapy without concurrent radiotherapy is reported.

## Case presentation

**Figure 1:**
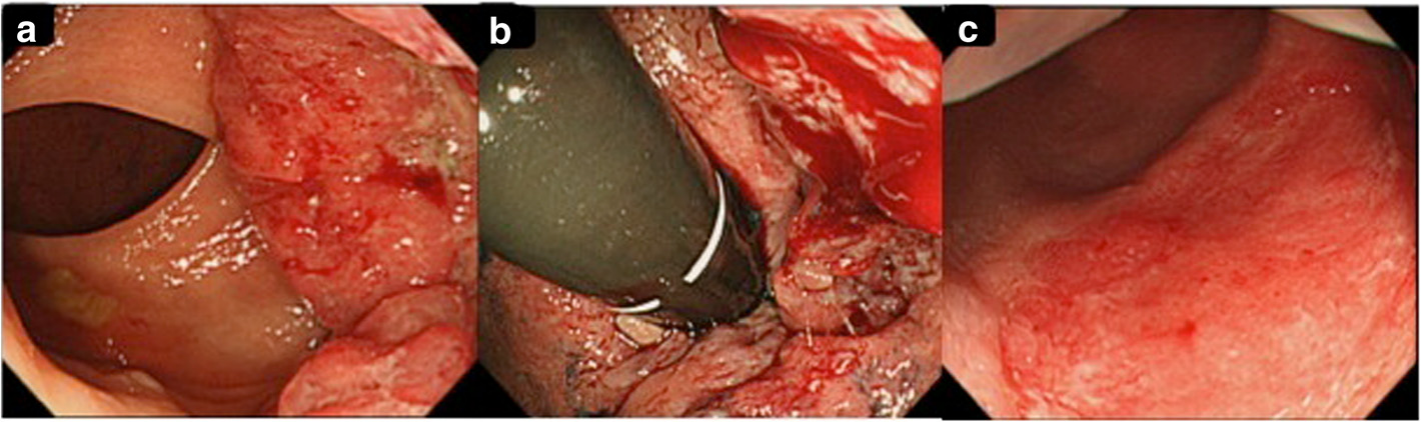
**Colonoscopy images. (a)** Colonoscopy imaging shows a 3 cm in length, type 2 rectal cancer **(b)** that invades to the dentate line. **(c)** Repeated colonoscopy after chemotherapy shows an excellent response with only injected mucosal scar in the area of the previously identified rectal cancer.

**Figure 2:**
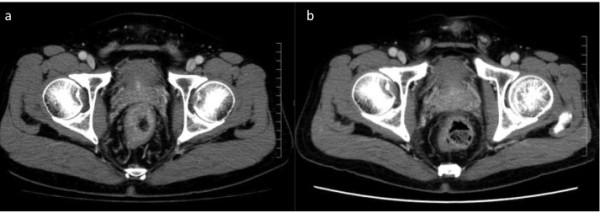
**Computed tomography (CT) images. (a)** CT imaging reveals rectal wall thickening and a regional lymph node metastasis in the mesorectum. **(b)** CT scan after chemotherapy demonstrates no rectal wall thickening and no mesorectal lymph node metastasis. CT, computed tomography.

A 53-year-old man was referred to Shiga University of Medical Science hospital, Shiga, Japan, complaining of bloody stool. The patient was diagnosed as having a 3 cm in length, type 2 *K-ras* wild type rectal cancer, 2 cm from the anal verge (Figure 1a) that invaded to the dentate line (Figure 1b) on screening colonoscopy. Computed tomography (CT) revealed rectal wall thickening and a regional lymph node metastasis in the mesorectum (Figure 2a). Advanced stage, low-lying rectal cancer was diagnosed. We usually perform abdominoperineal resection (APR) for advanced rectal cancer located in the anal canal as in this case. However, the patient was not willing to undergo APR.

Previously, Canda *et al*. reported that neoadjuvant CRT may adversely affect anorectal function [5]. It has also been reported that Cmab and Pmab-containing chemotherapy enables early tumor shrinkage [4]. For these reasons, neoadjuvant chemotherapy (NAC) with mFOLFOX6 and Pmab was performed every 2 weeks with the patient’s written, informed consent. The patient received a total of four cycles of chemotherapy over 2 months.

**Figure 3:**
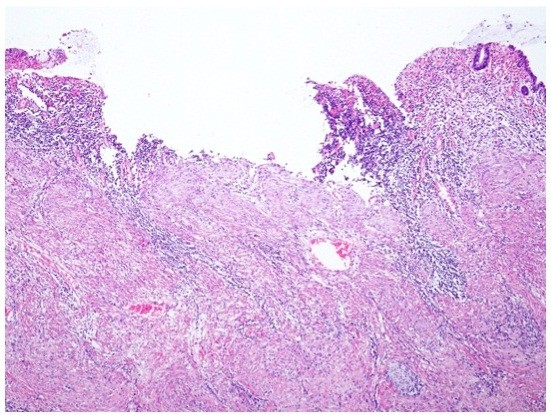
No residual carcinoma is found on histologic examination.

Repeated colonoscopy after chemotherapy showed an excellent response with only injected mucosal scar in the area of the previously identified rectal cancer (Figure 1c). The results of the biopsies were negative for cancer cells. Additionally, CT scan demonstrated no rectal wall thickening and no mesorectal lymph node metastasis (Figure 2b). The patient underwent total ISR with a handsewn coloanal anastomosis, total mesorectal and bilateral pelvic lymph node dissection, and temporary loop ileostomy 3 weeks after completion of NAC. No residual carcinoma was found (Figure 3), 12 non-involved lymph nodes were identified, and all margins were negative for cancer on histologic examination.

5-FU-based adjuvant chemotherapy after neoadjuvant CRT followed by surgery is the standard treatment for clinical stage III rectal cancer [1]. For this reason, the patient received adjuvant chemotherapy with tegafur-uracil (UFT) and leucovorin administration for 3 months after surgery. Three months after surgery, the patient is alive without evidence of recurrence of cancer.

## Discussion

Preoperative 5-FU-based concurrent CRT followed by surgery is the standard strategy for advanced rectal cancer in Western countries [1]. However, it has been suggested that preoperative CRT is strongly associated with postoperative complications, including anastomotic leakage [6] and poor anal function, after low anterior resection and ISR [3]. It has been reported that preoperative CRT also increases late complications such as sexual and voiding dysfunctions [7], intestinal and defecation problems [8], and secondary carcinogenesis [9]. All of these have recently become issues in Western countries.

Recently, new anticancer agents have markedly improved the response rate and prognosis of unresectable and recurrent colorectal cancer. Oxaliplatin-based combination chemotherapy improves survival of colorectal cancer patients in the metastatic and adjuvant settings [10-12]. Oxaliplatin-containing ‘induction chemotherapy’ before CRT is associated with an objective response rate of up to 88% and rapid symptomatic improvement [13,14]. These regimens may also convert unresectable liver metastases to resectable ones [15,16]. It has been reported that bevacizumab (Bmab), an anti-vascular endothelial growth factor (VEGF) receptor antagonist, added to oxaliplatin-based chemotherapy, capecitabine plus oxaliplatin (XELOX), resulted in a high response rate for patients with colorectal liver metastases [17]. In Japan, a multicenter phase II trial of NAC with XELOX plus Bmab for locally advanced rectal cancer is now ongoing [18]. In addition, two retrospective studies have shown that the early dimensional reduction of target lesions after 6 weeks from the beginning of treatment is an indicator of sensitivity to Cmab [19,20]. Pmab, a fully human immunoglobulin G (IgG) anti-EGFR antibody, has been considered equally effective in patients with *K-ras* wild type refractory metastatic colorectal cancer. Cmab must be administered every week, while Pmab can be administered every 2 weeks.

In the neoadjuvant setting, surgery must be delayed for at least 1 month after the last Bmab-containing chemotherapy. However, it is not necessary to delay surgery after anti-EGFR-containing chemotherapy. Because of these reasons, we considered that preoperative mFOLFOX6 and Pmab chemotherapy should be effective for this case. Recently, Li *et al*. reported a case of advanced rectal cancer demonstrating a pathologic complete response after NAC with six cycles of FOLFOX7 [21]. This case is the first report in the English literature from an Asian country demonstrating a pathological complete response after NAC in a patient with low-lying advanced rectal cancer. In the present case, NAC was given for four cycles, but the appropriate period of NAC administration has not been determined. However, a pilot study demonstrated clear downstaging of primary colon cancer with only three cycles of NAC [22]. Another report showed that detection, by week 2 magnetic resonance imaging, of tumor shrinkage >10% in response to therapy with Cmab or Pmab for metastatic colorectal cancer represents an early indicator of clinical outcome because it is predictive of the prolongation of progression-free survival and overall survival [4]. We thought that if NAC was not effective, the patient would not be able to receive curative surgery because of disease progression. Therefore, we evaluated the efficacy of NAC after a short course (four cycles) of chemotherapy. We then decided to perform surgery because of the excellent response to NAC. The present case suggests that Pmab is a good candidate for NAC because of its earlier drug response. We consider NAC is a promising preoperative treatment for locally advanced rectal cancer instead of neoadjuvant CRT. However, there are no data about whether NAC with or without concurrent radiotherapy is effective against advanced rectal cancer. Further studies are needed.

## Conclusions

This case suggests that mFOLFOX6 and Pmab chemotherapy without irradiation may be an alternative therapy for patients with low-lying rectal cancer.

## Consent

Written informed consent was obtained from the patient for publication of this case report and any accompanying images. A copy of the written consent is available for review by the Editor-in-Chief of this journal.

## Abbreviations

5-FU: 5-Fluorouracil; APR: Abdominoperineal resection; Bmab: Bevacizumab; Cmab: Cetuximab; CRT: Chemoradiotherapy; CT: Computed tomography; EGFR: Endothelial growth factor receptor; IgG: Immunoglobulin G; ISR: Intersphincteric resection; MRI: Magnetic resonance imaging; NAC: Neoadjuvant chemotherapy; Pmab: Panitumumab; UFT: Tegafur-uracil; VEGF: Vascular endothelial growth factor; XELOX: Capecitabine plus oxaliplatin

## Competing interests

The authors declare that they have no competing interests.

## Authors’ contributions

HS drafted the manuscript. TS, EM, YE and TT helped to draft the manuscript. MI created Figure 3. All authors read and approved the final manuscript.
